# Early life maternal separation induces sex-specific antidepressant-like responses but has minimal effects on adult stress susceptibility in mice

**DOI:** 10.3389/fnbeh.2022.941884

**Published:** 2022-09-12

**Authors:** Brittany J. Baugher, Benjamin D. Sachs

**Affiliations:** Department of Psychological and Brain Sciences, Villanova University, Villanova, PA, United States

**Keywords:** early life stress, sex differences, stress susceptibility, behavior, mouse model

## Abstract

Early life stress is known to increase the risk of depression and anxiety disorders, which are highly prevalent conditions that disproportionately affect women. However, the results of preclinical studies have been mixed, with some work suggesting that early life stress promotes anxiety-like behavior and/or increases susceptibility to subsequent stressors, and other research suggesting that early life stress reduces anxiety-like behavior and/or confers resilience to subsequent stress exposure. It is likely that factors such as sex and the timing and severity of early life and adult stress exposure dictate whether a particular early life experience promotes adaptive vs. maladaptive behavior later in life. Most work in this area has focused exclusively on males, but several sex differences in the effects of early life stress on subsequent stress susceptibility have been reported. The current study examined the impact of early life maternal separation on susceptibility to behavioral alterations induced by 3 days of variable stress in adulthood in male and female c57BL6 mice. Our results indicate that 3 days of adult stress is sufficient to increase anxiety-like behavior in several paradigms and to increase immobility in the forced swim test. In contrast, a history of maternal separation reduces anxiety-like behavior in several tests, particularly in males. These findings could contribute to our understanding of sex differences in mental illness by demonstrating that males are more likely than females to display adaptive responses to mild early life stressors.

## Introduction

Early life adversity has been linked to many negative consequences in adulthood, including an increased risk of depression, anxiety, and substance use disorders (Bale et al., 2010; Teicher et al., 2021). Several preclinical models of early life stress (ELS) have been developed and studied to provide insight into the neurobiological processes through which ELS contributes to mental illness. However, the relevance of existing models of ELS, such as early life maternal separation (MS), remains controversial, as MS has been reported to exert both positive or negative effects (Murthy and Gould, 2018). For example, several studies have reported that MS induces depression- and/or anxiety-like behaviors in rodents (Kalinichev et al., 2002; Romeo et al., 2003; Parfitt et al., 2004; Murgatroyd et al., 2009), but other studies indicate that MS alone does not reliably produce depression- or anxiety-like behavior (Millstein and Holmes, 2007; Parfitt et al., 2007). Similarly, multiple studies have shown that MS can lead to a long-lasting increase in corticosterone secretion following adult stress (Kalinichev et al., 2002; Parfitt et al., 2004; Murgatroyd et al., 2009; Sachs et al., 2013), although not all studies have reported this effect (Parfitt et al., 2007; Hsiao et al., 2016). MS-induced augmentation of corticosterone responses to adult stressors could have important implications for adult stress susceptibility, and several groups have reported that MS confers increased vulnerability to social defeat stress (SDS) in adulthood (Pena et al., 2017; Hultman et al., 2018). In contrast, another early life stressor, exposure to limited bedding and nesting material, has been reported to confer resilience to SDS in adulthood (Santarelli et al., 2017). Research in non-human primates has demonstrated that early life stress inoculation increases stress resistance in primates (Parker et al., 2006; Lyons and Parker, 2007; Lyons et al., 2010). These conflicting findings could result from differences in the ELS model or in differences in the strains or species of animals used (the studies reporting ELS-induced susceptibility used c57BL6/J mice, whereas the rodent study reporting ELS-induced resilience used Balb/c animals). Nonetheless, it will be important to test the effects of ELS on susceptibility to stressors other than SDS in rodents and to extend these types of studies to include females.

Examining potential sex differences is important in part due to the clinically observed sex differences in the incidence of mental illness, with women being nearly twice as likely as men to suffer from major depression or anxiety disorders (Kessler et al., 2012). The basis for these sex differences in psychiatric outcomes remains largely unknown, but preclinical studies examining neurobiological and behavioral responses to stress in males and females could improve our understanding of these sex differences. For example, researchers have developed a sub-chronic stress paradigm that induces more profound anxiety- and depression-like behavioral alterations in females compared to males (LaPlant et al., 2009; Hodes et al., 2015), an effect that appears to be driven in part by sex differences in transcriptional responses to stress (Hodes et al., 2015). However, several similar models of sub-chronic stress have been shown to induce largely similar behavioral responses in males and females despite sex differences in molecular stress responses (Eck et al., 2020; Baugher et al., 2022). The precise features of stress that lead to sex-specific responses, and the types of behavioral processes that are differentially impacted by stress in males and females still largely remain to be determined.

While sub-chronic stress paradigms lasting 5 or 6 days are consistently reported to induce behavioral phenotypes in at least one sex (Hodes et al., 2015; Baugher et al., 2022), 3 days of variable stress exposure has generally been reported to be insufficient to alter behavior (Hodes et al., 2015; Duque-Wilckens et al., 2022). However, experimental manipulations have been shown to confer vulnerability to 3 days of stress exposure in mice. For example, one recent study reported that female mice exposed to 3 days of stress in adulthood following ELS (MS plus early weaning) have reduced sucrose preference compared to mice exposed to 3 days of stress in adulthood without a history of ELS. Importantly, neither ELS nor 3 days of adult stress alone led to reductions in sucrose preference (Duque-Wilckens et al., 2022). The current study was designed to determine whether MS influences susceptibility to an abbreviated 3-day version of the 5-day variable stress paradigm (5DS) our lab has recently published (Baugher et al., 2022). The 5DS protocol has been applied in studies with handled controls and with undisturbed controls. Largely similar “anxiety-like” or “depression-like” behavioral alterations were observed regardless of handling in several tests [i.e., the elevated plus maze (EPM) and forced swim test (FST)], but 5DS-induced increases in anxiety-like behavior in the light-dark emergence (LDE) test were only observed in comparison to undisturbed controls, not in comparison to handled controls (Baugher et al., 2022). The current study left control animals undisturbed.

## Materials and methods

### Subjects

This study used a total of 77 c57BL/6 male and female mice that were bred in the Sachs Lab at Villanova University. The mice were housed in a temperature-controlled room on a 12 h light-dark cycle and provided food and water *ad libitum*. All procedures were covered by a protocol approved by Villanova University’s Institutional Animal Care and Use Committee.

### Maternal separation

**FIGURE 1:**
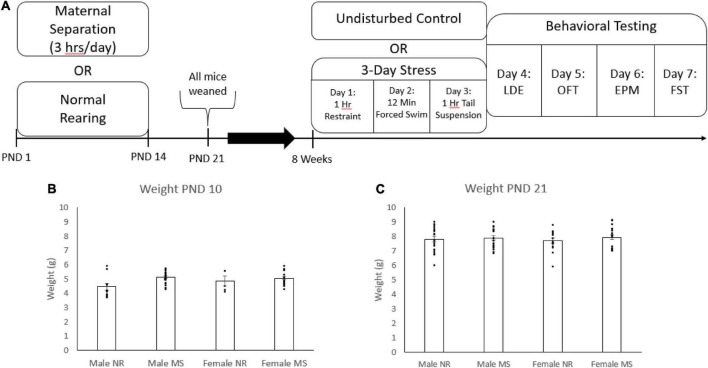
The timeline of stressors and behavioral tests throughout the study **(A)**. Mice were weighed at PND 10 **(B)** and PND 21 **(C)**. Error bars represent the standard error from the mean, and individual data points are included. For PND 10, *N* = 10 for male NR, 21 for male MS, 7 for female NR, and 20 for female MS. At PND 21 *N* = 21 for male NR, 21 for male MS, 15 for female NR, and 20 for female MS.

Mice in the MS-exposed litters were separated from their dams for 3 h each day during the light period. During the separation period, the pups were placed in plastic holding containers where they remained in contact with their littermates. The separation began on post-natal day (PND) 1 and continued every day until PND 14. Control litters remained in normal rearing (NR) conditions and were left undisturbed until weaning. All mice were weaned at PND 21 (Figure 1A).

### Three-day stress

When mice reached 8 weeks of age, half were exposed to a 3 day stress paradigm (3DS) while the other half were left as undisturbed controls. On day 1, the 3DS mice were exposed to 1 h of restraint inside a ventilated 50 ml conical tube. Day 2 consisted of a 12-min forced swim in 25° C water. On day 3, the 3DS mice were suspended by their tails for 1 h. Behavioral testing began on day 4, approximately 24 h after the final stressor. Control animals were left undisturbed until behavioral testing.

### Light-dark emergence

Mice were tested in the LDE test as we have described previously (Baugher et al., 2022). ANYmaze animal tracking software was used to measure time, distance, and entries in the light compartment of the box, as well as latency to enter the light compartment for each mouse.

### Open field test

Each mouse was also evaluated in the open-field test (OFT). Each mouse was placed in a 40 × 40 cm well-lit activity chamber for 20 min. The movement and location of the mouse were recorded and analyzed using ANYmaze software. The total distance traveled, the distance traveled in the center, the number of entries to the center, and the time spent in the center region were measured.

### Elevated plus-maze

The EPM was conducted using ANYmaze animal tracking software as described previously (Baugher et al., 2022). Each mouse was individually placed in one of the closed arms of the EPM and allowed to explore for a total of 5 min. During this time, the mouse’s location and movements were recorded to determine the time spent and distance traveled in the open arms and the closed arms, and the latency to enter the open arms.

### Forced swim test

The FST was performed as described previously (Baugher et al., 2022). Mice were placed in a 4L glass beaker filled with 2,500 ml of 25°C water for a total of 6 min. The mice were recorded from above using a camera suspended above the beaker. ANYmaze software was used to measure the amount of time that each mouse spent immobile and the number of discrete immobile episodes as well as the total distance traveled and the latency to the first immobile episode.

### Experimental timeline

The timeline of the experiment is shown in Figure 1A. Briefly, mice were subjected to maternal separation or normal rearing during the first 14 days of life, as described above. Mice were weighed on PND 10 and PND 21 and then were weaned at 3 weeks of age. Mice were subsequently subjected to either 3DS or left as controls starting when they reached 8 weeks of age as described above. Starting the day after the end of 3DS, mice were tested in the LDE (1d after 3DS), the OFT (2d after 3DS), the EPM (3d after 3DS), and the FST (4d after 3DS) on consecutive days.

### Statistical analyses

Data were analyzed using full factorial three-way ANOVAs (2 × 2 × 2) with the following between-subjects factors: sex (male vs. female) × ELS (MS vs. NR) × adult stress (3DS vs. control). Statistical analyses were performed using SPSS, and *p*-values of less than 0.05 were considered significant. One outlier from the male NR control group was greater than 3 SD below the mean on all measures and scored at zero on measures of time and distance in the light area. Therefore, this mouse was removed before analysis of the LDE test. Following the initial analyses, additional two-way adult stress by early life stress ANOVAs stratified by sex were run for each dependent variable.

## Results

### Weights at post-natal day 10 and post-natal day 21

Mice in the normal rearing condition and the maternal separation condition were weighed at PND 10 and PND 21 (Figures 1B,C). Due to researcher error, weight data was missing for a portion of the normal rearing group during the PND 10 measurement. Mice in the MS condition weighed significantly more than the mice in the NR condition at PND 10 [*F*_(1, 58)_ = 5.45, *p* = 0.023, Figure 1B], but not PND 21. There were no significant differences between sex at either time point, and no interactions between sex and ELS condition.

### Light-dark emergence

**FIGURE 2:**
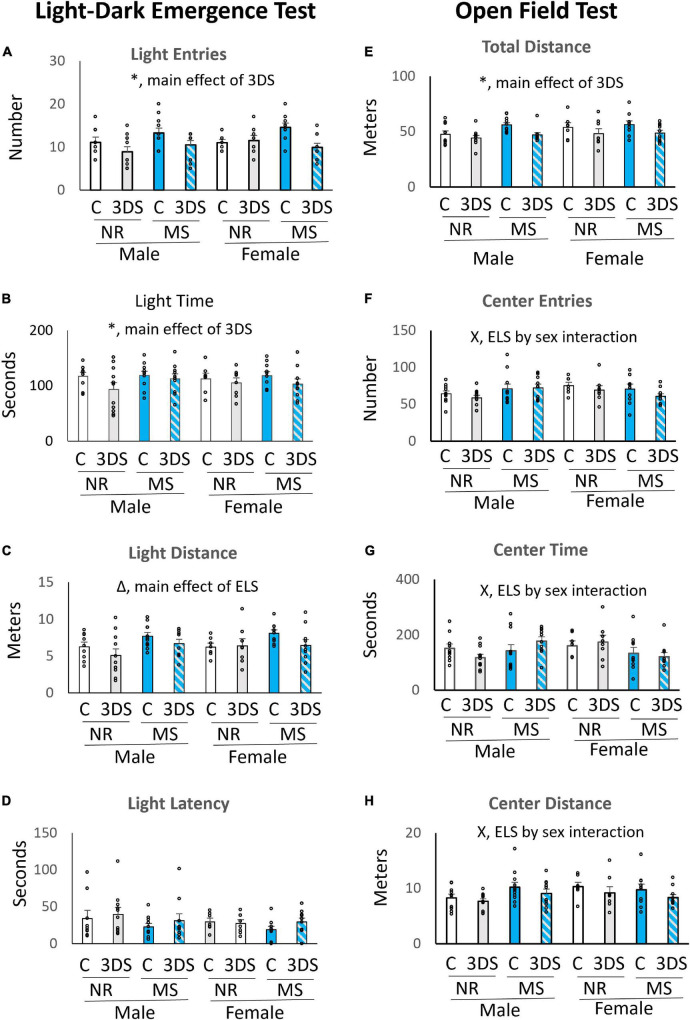
The effects of maternal separation and 3 days of variable stress on behavior in the light-dark emergence test and open field test. For the light-dark emergence test the average number of entries into the light chamber **(A)**, time spent in the light chamber **(B)**, distance traveled in the light chamber **(C)**, and latency until the first entry into the light chamber **(D)** are plotted. For the open field test the average total distance traveled **(E)**, number of entries into the center **(F)**, time spent in the center **(G)**, and distance traveled in the center **(H)** are plotted. Error bars reflect the standard error of the mean. Each individual data point is shown. NR, stands for normal rearing; MS, stands for maternal separation; C, stands for control; and 3DS stands for 3 days of stress exposure. For the open field test, *N* = 10 for male NR control, 11 for male NR 3DS, 11 for male MS control, 10 for male MS 3DS, 7 for female NR control, 8 for female NR 3DS, 10 for female MS control, and 10 for female MS 3DS. In the light-dark emergence test, one outlier was excluded from the Male NR control group leaving a sample size of *N* = 9. All other group sizes were identical. *Denotes a main effect of adult stress, Δ denotes a main effect of early life stress, and X denotes an early life stress by sex interaction by three-way ANOVA.

The LDE revealed a significant main effect of adult stress on light entries [*F*_(1, 75)_ = 9.48, *p* = 0.003, Figure 2A] and light time [*F*_(1, 75)_ = 4.28, *p* = 0.042, Figure 2B]. Mice in the control group spent more time in the light area and entered the light more times than mice in the 3DS group. The 3DS group also traveled slightly less distance in the light area than mice with no adult stress exposure, but this effect was not statistically significant [*F*_(1, 75)_ = 3.79, *p* = 0.056, Figure 2C]. For the measure of light distance, there was a significant main effect of ELS [*F*_(1, 75)_ = 6.66, *p* = 0.012, Figure 2C], with mice in the MS condition traveling further in the light area than mice in the control group. However, no significant main effects of ELS were observed in the number of light entries or the amount of time spent in the light compartment (Figures 2A,B). The measure of latency to enter the light compartment did not differ between groups (Figure 2D). No significant interactions were examined on any of the LDE measures when the sexes were analyzed together during the initial analyses.

When data were stratified by sex and analyzed by separate two-way ANOVAs for males and females, a significant interaction between ELS and adult stress was observed for the number of light entries for females [*F*_(1, 34)_ = 7.304, *p* = 0.011, Figure 2A], but no interaction was observed in males. Adult stress did not significantly impact the number of light entries in animals without a history of MS, but MS-exposed animals that were not exposed to 3DS entered the light area more than MS-exposed mice that were also exposed to 3DS.

### Open-field test

In the OFT, mice in the 3DS group traveled significantly less distance overall than the adult control group when sexes were examined together [*F*_(1, 76)_ = 10.54, *p* = 0.002, Figure 2E]. For the measure of center entries, there was a significant interaction between sex and ELS condition [*F*_(1, 76)_ = 5.73, *p* = 0.019, Figure 2F] in which females in the MS group were less likely to enter the center than control females, whereas males in the MS group were more likely to enter the center area than control males. This interaction between sex and ELS condition was also significant for the measures of center time [*F*_(1, 76)_ = 6.9, *p* = 0.011, Figure 2G] and center distance [*F*_(1, 76)_ = 4.7, *p* = 0.034, Figure 2H]. There was a trend toward an effect of adult stress condition on center distance [*F*_(1, 76)_ = 3.74, *p* = 0.057, Figure 2H], where the control group traveled a greater distance in the center than the group exposed to 3DS, but this did not reach statistical significance. There was also a trend toward a three-way ELS by sex by adult stress interaction for the amount of time spent in the center [*F*_(1, 76)_ = 3.5, *p* = 0.065, Figure 2G], but this effect did not reach significance either.

During further analyses stratified by sex, a two-way ANOVA revealed a significant interaction between ELS and adult stress on center time in males [*F*_(1, 41)_ = 4.29, *p* = 0.045], but no such interaction was observed in females. For this interaction in males, 3DS slightly reduced center time in mice with no history of MS. However, in MS-exposed animals, 3DS slightly increased center time. No other significant ELS by adult stress interactions were observed in either sex.

### Elevated plus-maze

**FIGURE 3:**
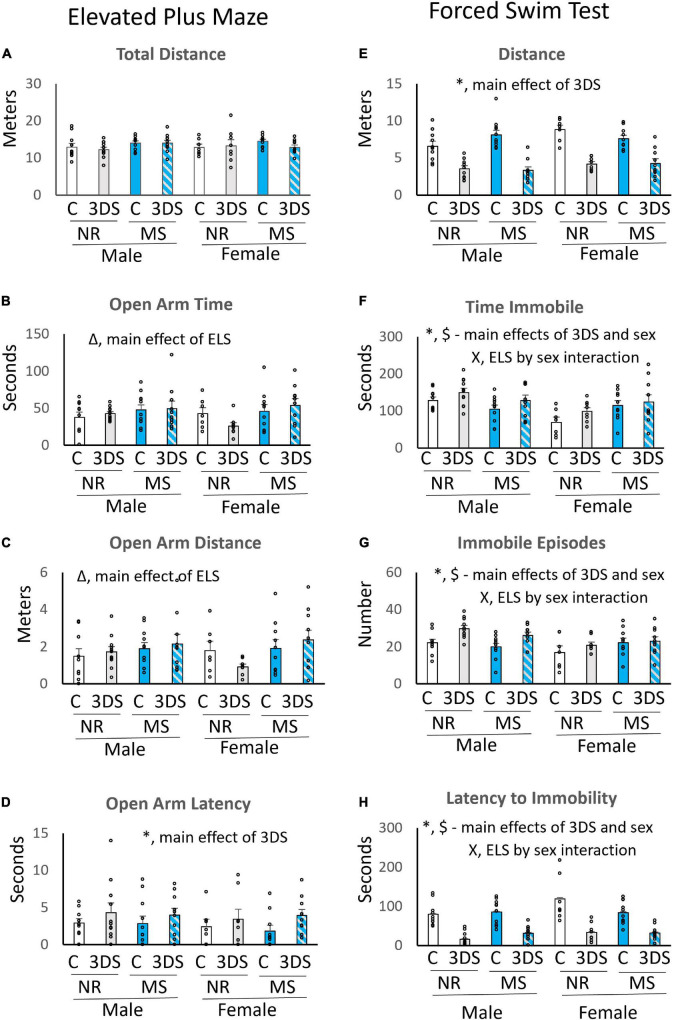
The effects of maternal separation and 3 days of variable stress on behavior in the elevated plus maze and forced swim test. For the elevated plus maze the average total distance traveled **(A)**, time spent in the open arms **(B)**, distance traveled in the open arms **(C)**, and latency until the first entry into an open arm **(D)** are plotted. For the forced swim test the average total distance traveled **(E)**, time spent immobile **(F)**, number of immobile episodes **(G)**, and latency until the first immobile episode **(H)** are plotted. Error bars reflect the standard error of the mean. Each individual data point is shown. NR, stands for normal rearing; MS, stands for maternal separation; C, stands for control; and 3DS stands for 3 days of stress exposure. *N* = 10 for male NR control, 11 for male NR 3DS, 11 for male MS control, 10 for male MS 3DS, 7 for female NR control, 8 for female NR 3DS, 10 for female MS control, and 10 for female MS 3DS. *Denotes a main effect of adult stress, Δ denotes a main effect of early life stress, $ denotes a main effect of sex, and X denotes an early life stress by sex interaction by three-way ANOVA.

In the EPM, there were no group differences in total distance traveled (Figure 3A). However, main effects of ELS were observed for the measures of time in the open arms [*F*_(1, 76)_ = 5.45, *p* = 0.022, Figure 3B] and distance in the open arms [*F*_(1, 76)_ = 4.49, *p* = 0.038, Figure 3C]. The mice exposed to MS spent more time and traveled further in the open arms than the control group. Exposure to 3DS significantly increased the latency to enter the open arm [*F*_(1, 76)_ = 4.36, *p* = 0.04, Figure 3D], but 3DS did not impact any of the other measures. When the sexes were examined separately, no additional ELS by adult stress interactions were observed in either sex.

### Forced swim test

As expected, a main effect of adult stress was found on all measures examined in the FST. The mice exposed to 3DS swam a shorter distance [*F*_(1, 76)_ = 113.104, *p* < 0.001, Figure 3E], spent more time immobile [*F*_(1, 76)_ = 5.253, *p* = 0.025, Figure 3F], had more immobile episodes [*F*_(1, 76)_ = 9.902, *p* = 0.002, Figure 3G], and had a shorter latency to the first immobile episode [*F*_(1, 76)_ = 91.362, *p* < 0.001, Figure 3H] than the control group. A significant main effect of sex was also observed for distance, with females traveling a greater distance than males [*F*_(1, 76)_ = 4.765, *p* = 0.032, Figure 3E]. In addition, a three-way interaction between sex, ELS, and adult stress was found in the measure of swimming distance [*F*_(1, 76)_ = 4.22, *p* = 0.044, Figure 3E]. Significant main effects of sex on time immobile [*F*_(1, 76)_ = 8.28, *p* = 0.005, Figure 3F], immobile episodes [*F*_(1, 76)_ = 6.33, *p* = 0.014, Figure 3G], and immobility latency [*F*_(1, 76)_ = 4.46, *p* = 0.038, Figure 3H] were also observed, with males spending more time immobile than females, having more immobile episodes, and a shorter latency to first immobile episode. There were also significant interactions between sex and ELS for immobility time [*F*_(1, 76)_ = 10.10, *p* = 0.002, Figure 3F], the number of immobile episodes [*F*_(1, 76)_ = 0.03, *p* = 0.028, Figure 3G], and immobility latency [*F*_(1, 76)_ = 4.52, *p* = 0.037, Figure 3H]. For these sex by ELS interactions, MS tended to reduce immobility time and episodes while increasing immobile latency in males, but it tended to increase immobile time and episodes while reducing latency to immobility in females.

When the data were analyzed separately by sex, the effects of ELS on immobility time in males did not quite reach significance [*F*_(1, 41)_ = 4.077, *p* = 0.051], but a history of MS did significantly increase time spent immobile in females [*F*_(1, 34)_ = 5.681, *p* = 0.023]. No other significant effects of ELS were observed within either sex, and no significant ELS by adult stress interactions were observed in either sex.

## Discussion

The current study adds to a growing literature investigating the impact of early life experience on long-term behavioral outcomes. Our results demonstrate that the MS procedure used here leads to several significant antidepressant- or anxiolytic-like behavioral alterations in c57BL6 mice, particularly in males. These results did not support our hypothesis that MS would increase susceptibility to depression- and anxiety-like alterations induced by 3DS. Our hypothesis had been based largely on a prior study in which we and our collaborators had shown that this MS procedure increased susceptibility to social avoidance behavior induced by SDS (Hultman et al., 2018). The reasons why MS increases susceptibility to SDS but not 3DS remain unclear but could result from qualitative differences in the type of adult stress encountered (social vs. not-social). Alternatively, these differences could stem from the fact that different behavioral outcome measures were used for the 3DS (LDE, OFT, EPM, and FST) vs. the SDS (social interaction) studies.

There is considerable variability in the reported effects of ELS on anxiety- and depression-like behavior. Indeed, as stated above, ELS-induced increases in anxiety- and depression-like behavior have been reported (Kalinichev et al., 2002; Romeo et al., 2003; Parfitt et al., 2004; Murgatroyd et al., 2009), but other studies indicate that MS alone does not reliably produce depression- or anxiety-like behavior (Millstein and Holmes, 2007; Parfitt et al., 2007). Still other studies have reported MS-induced reductions in anxiety (McIntosh et al., 1999; Eklund and Arborelius, 2006). The current results suggest that a history of MS can induce anxiolytic-like effects that can partially counteract the consequences of adult stress. Therefore, the current findings are consistent with the stress inoculation hypothesis, which argues that brief ELS exposure can protect against negative consequences of future stressors (Daskalakis et al., 2013). Several prior studies in rodents have also supported the stress inoculation hypothesis by reporting protective effects of brief or mild ELS (Hsiao et al., 2016; Calpe-Lopez et al., 2022). However, other studies have suggested that similar ELS procedures promote susceptibility to subsequent stressors (Pena et al., 2017; Hultman et al., 2018; Duque-Wilckens et al., 2022). The reasons for these disparate outcomes following ELS and adult stress are not known, but they could involve unidentified genetic (or other) factors, consistent with the three-hit-hypothesis of vulnerability vs. resilience (Daskalakis et al., 2013).

Given the clinical observation that ELS increases the risk of mental illness, it is possible that the MS procedure used here is not severe enough to model the types of early life adversity associated with negative outcomes. Indeed, when separated from the dam, the pups were still with their littermates, which may have reduced the amount of stress experienced during the separation period. In addition, the dams may have offset the stress experienced by the pups by increasing the amount of grooming and care following the separation. Increased maternal care (grooming and licking of pups) has been shown to reduce signs of anxiety in mice (Liu et al., 1997). Brief MS has been shown to increase grooming upon reunion of pups and dams (Levine et al., 1956), and it appears that the benefits of increased grooming following MS outweighed any detrimental effects of separation. Consistent with this, our data indicate that MS did not reduce the body weights of mice at the time of weaning on PND 21, thus suggesting that their care was not significantly impaired. Future research is needed to determine if increasing the severity of ELS and/or limiting compensatory maternal care would promote negative outcomes following MS.

The current results are not consistent with a recent report demonstrating that the combination of early life MS with early weaning increases susceptibility to adult-stress-induced changes in the sucrose preference test (Duque-Wilckens et al., 2022). There are many procedural differences between these studies that likely explain the apparent discrepancy. For example, the prior study isolated the pups from their littermates during MS and MS was performed over 17 days instead of the 14 days used here. The intensity of ELS in the previous study was likely further magnified by the early weaning on day 17. Importantly, even the more severe ELS model did not have any significant effects on sucrose preference on its own (Duque-Wilckens et al., 2022), but it did predispose animals to sucrose preference reductions following an adult stressor.

There are also differences in the intensity of the adult 3DS paradigm used in the current study compared to the recent publication reporting ELS-induced increases in adult stress susceptibility. Specifically, the recently published study consisted of 10 min of restraint on day 1, 10 min of tail suspension on day 2, and 10 min of foot shocks on day 3 (Duque-Wilckens et al., 2022). The current study used 1 h of restraint and tail suspension and 12 min of forced swimming. Thus, the adult stress paradigm used here was likely more severe. Consistent with this, unlike several prior studies using 3-day variable stress paradigms in which no significant behavioral effects of 3DS were reported (LaPlant et al., 2009; Hodes et al., 2015; Zhang et al., 2018; Duque-Wilckens et al., 2022), the 3DS paradigm used here was sufficient to impact behavior in the LDE, FST, and the OFT, but not the EPM (other than the latency measure). In comparison, our prior work revealed that the slightly longer 5DS paradigm led to similar phenotypes as 3DS in the FST and LDE, but it also promoted anxiety-like behavior in both males and females in the EPM (Baugher et al., 2022), unlike the 3DS version. This suggests, as one might expect, that 3DS is less severe than 5DS, but further reductions in adult stress severity could prove beneficial to identify factors that increase vulnerability.

The current results suggest that MS can promote antidepressant-like or anxiolytic-like effects in several behavioral measures, but several interesting sex differences were also noted in which some of the effects of MS appeared more beneficial in males than females. For example, in the OFT, an MS by sex interaction was observed in which MS tended to reduce anxiety in males but increase it in females. Although hormonal data was not collected in this study, it is important to consider that the female estrous cycle may be contributing to the sex differences observed here (ter Horst et al., 2012). Similarly, in the FST, MS reduced immobility in the males but increased it in the females. Admittedly, the interpretation of FST data is highly controversial, with some arguing that it measures depression-like behavior (i.e., behavioral despair) (Porsolt et al., 1977), others arguing that it measures anxiety-like behavior (Anyan and Amir, 2018), and others arguing that it measures stress coping strategies (Commons et al., 2017). The current study’s design, in which forced swimming is used as both a stressor and a behavioral output measure, further complicates the interpretation of FST data. Indeed, due to their prior experience swimming, the 3DS groups would be expected to exhibit increased immobility (which they do) independent of behavioral despair or anxiety. Indeed, it is possible that a history of swimming allows mice to learn to adopt a passive stress coping strategy. Nonetheless, the fact that forced swimming behavior depends on interactions between sex and ELS (and not just 3DS alone) suggest that mice of different sexes or stress histories adopt passive coping strategies to a different extent. Whether the sex and ELS-induced differences in immobility result exclusively from alterations in stress coping strategies, or whether differences in despair, anxiety, and/or learning also contribute remains unclear. Regardless, dysfunction in any of these processes could have important implications for mental health.

There have been reports that behavior in the EPM reflects primarily exploratory drive in females and primarily anxiety in males (Fernandes et al., 1999). Consequently, similar levels of open arm time in this test may be observed despite different levels of anxiety. Males and females have been reported to exhibit distinct manifestations of fear/anxiety-related behaviors on other tasks, such as fear conditioning (Gruene et al., 2015), but the extent to which the dependent variables measured in the tests used here reflect similar behavioral processes in males and females remains incompletely understood, and should be the focus of future research.

Our within-sex analyses revealed only two significant interactions between ELS and adult stress: light entries in the LDE in females and center time in the OFT in males. In the LDE in females, mice exposed to MS and 3DS showed more anxiety-like behavior than those exposed to MS alone. However, the number of light entries in the MS-3DS group was not significantly lower than control animals, so it would be somewhat misleading to argue that the MS-3DS group exhibited anxiety-like behavior. Rather, MS appeared to reduce anxiety-like behavior, but 3DS reversed this effect. An ELS by adult stress interaction was also observed in males in the OFT. In NR males, 3DS appeared to reduce the time spent in the center, but MS appeared to protect against this anxiogenic effect of 3DS. This finding seems to indicate a predictive adaptive response in which exposure to stress in both early life and adulthood led to better outcomes than adulthood stressors alone, at least for this measure.

Overall, our results are consistent with prior studies suggesting that MS alone does not provide a reliable model of ELS-induced anxiety- or depression-like behaviors. Furthermore, the stress-susceptibility promoting effects of MS (Pena et al., 2017; Hultman et al., 2018) do not appear to extend to the 3DS model of sub-chronic variable stress. In fact, the MS model used here actually produced antidepressant-like or anxiolytic-like effects in several commonly used behavioral tests, although sex differences were observed. Whether the behavioral improvements that we observed following MS in the current study result from increased maternal care or from some other factor, our findings reinforce the idea that males and females are differentially sensitive to environmental changes in early life that could have important implications for adult mental health.

## Data availability statement

The raw data supporting the conclusions of this article will be made available by the authors, without undue reservation.

## Ethics statement

The animal study was reviewed and approved by the Villanova University Institutional Animal Care and Use Committee.

## Author contributions

BS and BB conceptualized and designed the study. BS wrote the first draft of the manuscript and performed data analysis. BB performed the experiments, collected and analyzed the data, and revised the manuscript. Both authors contributed to the article and approved the submitted version.
